# Neural processing of food and emotional stimuli in adolescent and adult anorexia nervosa patients

**DOI:** 10.1371/journal.pone.0191059

**Published:** 2018-03-26

**Authors:** Stefanie Horndasch, Julie Roesch, Clemens Forster, Arnd Dörfler, Silja Lindsiepe, Hartmut Heinrich, Holmer Graap, Gunther H. Moll, Oliver Kratz

**Affiliations:** 1 Department of Child and Adolescent Mental Health, University of Erlangen-Nuremberg, Erlangen, Germany; 2 Department of Neuroradiology, University of Erlangen-Nuremberg, Erlangen, Germany; 3 Institute for Physiology and Pathophysiology, University of Erlangen-Nuremberg, Erlangen, Germany; 4 Heckscher Klinikum, Munich, Germany; 5 Department of Psychosomatic Medicine and Psychotherapy, University of Erlangen-Nuremberg, Erlangen, Germany; Harvard Medical School, UNITED STATES

## Abstract

**Background:**

A constant preoccupation with food and restrictive eating are main symptoms of anorexia nervosa (AN). Imaging studies revealed aberrant neural activation patterns in brain regions processing hedonic and reward reactions as well as–potentially aversive–emotions. An imbalance between so called “bottom-up” and “top-down” control areas is discussed. The present study is focusing on neural processing of disease-specific food stimuli and emotional stimuli and its developmental course in adolescent and adult AN patients and could offer new insight into differential mechanisms underlying shorter or more chronic disease.

**Methods:**

33 adolescents aged 12–18 years (15 AN patients, 18 control participants) and 32 adult women (16 AN patients, 16 control participants) underwent functional magnetic resonance imaging (fMRI, 3T high-field scanner) while watching pictures of high and low-calorie food and affective stimuli. Afterwards, they rated subjective valence of each picture. FMRI data analysis was performed using a region of interest based approach.

**Results:**

Pictures of high-calorie food items were rated more negatively by AN patients.

Differences in activation between patients and controls were found in “bottom up” and “top down” control areas for food stimuli and in several emotion processing regions for affective stimuli which were more pronounced in adolescents than in adults.

**Conclusion:**

A differential pattern was seen for food stimuli compared to generally emotion eliciting stimuli. Adolescents with AN show reduced processing of affective stimuli and enhanced activation of regions involved in “bottom up” reward processing and “top down” control as well as the insula with regard to food stimuli with a focus on brain regions which underlie changes during adolescent development. In adults less clear and less specific activation differences were present, pointing towards a high impact that regions undergoing maturation might have on AN symptoms.

## Introduction

Anorexia nervosa (AN) is a chronic mental disorder that often has its onset in adolescence. There is scientific evidence for changes in central nervous system function in AN and a “pathological neurocircuitry in AN” has been proposed [1]. Frequently, neural processing of disease-specific stimuli like those referring to food and nutrition has been examined. A meta-analysis of a total of nine fMRI studies on food processing in AN revealed greater activations in patients compared with controls in emotion-processing regions [2]. The authors conclude that food stimuli may be more emotionally arousing to AN patients. Indeed, there are studies which point to a mainly fear-driven neural processing of particularly high-calorie food stimuli in AN patients [3, 4]. By contrast, less amygdala, insula and hypothalamus activation has been found in AN patients when looking at similar food pictures [5], interpreted as a less active food reward system in AN. However, the meaning of increased–or, in some cases, decreased–activation of emotion-processing regions is still unclear. Those regions are involved in many aspects of emotional processing including that of stimuli with positive valence (see e.g. [6]).

In normal eating behavior, so called appetite-related “bottom-up” (BU, involving emotional and reward-related brain regions) and “top-down” (TD, cognitive control-related neural circuitry) responses to food stimuli are described that facilitate the maintenance of equilibrium between food intake and energy expenditure (see Table 1 for regions shown to be involved in BU and TD circuits). This equilibrium seems to be altered in eating disorders (ED) [7]. While several study results [8–10] suggest increased activity in the dorsolateral prefrontal cortex (DLPFC) in AN, possibly representing increased TD control, the results on the involvement of BU regions are not as unequivocal: either increased or blunted reward responses to food stimuli have been shown in AN [11–13]. A recent study looking at BU vs. TD processes during visual food processing in currently ill and recovered AN discovered not reduced, but increased BU activation in the caudate in both patient groups as well as higher activity in TD control areas [14].

**Table 1:** Examples for areas involved in “bottom-up” and “top-down” brain circuits.

Brain region	Study reference
**Bottom-up**	
lateral orbitofrontal cortex, putamen/anteroventral striatum, insula, thalamus	Frank et al., 2012
caudate, supplementary motor area, precentral gyrus	Brooks et al., 2011
caudate, striatum, hippocampus, amygdala, hypothalamus, cerebellum	Sanders et al., 2015
ventral striatum, cingulate cortex, putamen	Cowdrey et al., 2011
insula, ventral and dorsal striatum	Wagner et al., 2008
**Top-down**	
medial and lateral prefrontal cortex, anterior cingulate	Sanders et al., 2015
dorsolateral prefrontal cortex	Brooks et al., 2011; Frank et al., 2012
medial prefrontal cortex	Cowdrey et al., 2011; Uher et al., 2003; Uher et al., 2004
The insula is often considered a “hub” connecting bottom-up and top-down areas (Brooks et al., 2011)	

One key region which has emerged in recent studies in the context of disturbed eating behavior is the insula, particularly the anterior part [15]: enhanced insula activity which was modulated by hunger and calorie content of food stimuli was found in healthy participants [16]. In AN, hypo- as well as hyperactivations have been found: weight restored and currently underweight AN patients showed in pre- and postmeal conditions decreased insula activation towards food pictures [5, 17] and after sucrose and water administration [12]. This has been interpreted as AN patients being less prone to respond to the hedonic aspects of food [7]. Increased anterior insula activation during the presentation of food images in recovered and currently ill AN patients could be related to anticipation of the emotionally salient food stimuli [18, 19].

Of note, in these studies predominantly adult patients were investigated. Adolescence is a critical period for the development of EDs [20] and adolescent brains are still in a phase of maturation which might affect processing of particularly emotional but also of food stimuli. A study in typically developing adolescents comparing their responses when viewing high-calorie and low-calorie food images to those of adults showed a differential pattern in prefrontal, visual processing and reward-related brain regions [21]. Up to date, no study has examined potential neural differences in the processing of food stimuli between adolescent and adult AN patients and their matching control groups separately. Knowledge of such effects could be helpful for understanding the development and chronicity of aberrant eating behavior. Our aim therefore was to look at developmental effects in healthy females and those suffering from AN on the one hand and compare patients and control participants within the age groups on the other hand. A research question not addressed by most of the aforementioned studies is whether high- and low-calorie food stimuli are processed by adult and adolescent AN patients in a specific way. Activation differences have been observed dependent on calorie content of food in few studies [3, 22].

Affective symptoms are frequent and alterations in emotional processing are discussed in AN. E.g., lower activation of “affective” brain regions has been observed in the context of high alexithymia scores in AN patients when confronted with negative words [23]. By contrast, other studies using negative pictorial stimuli found no significant differences in brain activation between AN patients and healthy participants [24, 25]. To test if patients’ reduced preference and possibly aberrant brain responses to certain food may be associated with its caloric nature and mainly driven by the fear of weight gain or rather an impaired ability to experience mainly positive emotions in general, we included high- and low-calorie stimuli as well as non-disease specific emotion eliciting stimuli in our study.

Hypotheses:

H1: We hypothesize altered activation in BU and TD control areas in AN patients when confronted with food stimuli. Based on recent findings, we expect increased activity in BU as well as in TD areas (see Table 1) in AN patients and increased activation of the insula as a key region in that framework.
H2: In response to visual general emotion eliciting stimuli, we expect no significant differences regarding brain activation patterns between AN and control participants.
H3: Based on previous findings of developmental changes in processing of food images we hypothesize that regions which are sensitive towards changes in brain development (subcortical and frontal regions) are especially subject to alterations in adolescent AN patients as compared to typically developing adolescents.

## Materials and methods

### Participants

In total, 65 female participants were included: 33 adolescents (12–18 years) and 32 adults (19–40 years). The patient group consisted of 31 girls and women fulfilling the ICD-10 criteria for anorexia nervosa (ICD-10 F50.0, World Health Organization, 1993), the control group of 34 typically developing adolescent girls and healthy adult women in the same age range. AN patients were recruited at the Department of Child and Adolescent Mental Health and the Department of Psychosomatics and Psychotherapy at the University Clinic Erlangen at the beginning of an inpatient treatment program and received their diagnosis by an experienced psychiatrist or psychologist using the “Diagnostic System for Mental Disorders for Children and Adolescents (DISYPS-KJ)” (Döpfner et al., 2008) in adolescents and the “Structured Diagnostic Interview for Mental Disorders (DIPS)” (Schneider and Margraf, 2006) in adults. Control participants were recruited through advertisements and personal contact. Exclusion criteria for the control group were current or a history of psychiatric disorder and obesity. Further exclusion criteria for all participants were learning disabilities, severe neurological disease, a history of head trauma, visual impairments, metallic implants, claustrophobia and psychotropic medication other than selective serotonin reuptake inhibitors and atypical neuroleptics.

All participants completed a German version of the Eating Disorder Inventory-2 (EDI-2), which assesses ED psychopathology, is comprised of 91 items divided into 11 subscales and has an internal consistency (α) for clinical populations between .73 and .93) [26, 27].

Further the Beck Depression Inventory (BDI)-II was administered, the most widely used clinical instrument for detecting depression with a good internal consistency (α for clinical populations between .89 and .94, for non-clinical populations between .84 and .91) [28, 29].

Written informed consent was obtained from all participants and from the adolescents’ parents.

The Ethics Committee of the University Hospital of Erlangen gave approval for the study and it was conducted in accordance with the Declaration of Helsinki.

### Stimuli

12 colour photographs of high and low-calorie food on a light grey background were chosen from a pool of pictures which were selected from picture data bases and assessed by 10 healthy adults regarding their caloric content. Pictures of high and low-calorie food were matched according to colour, image density and area (in percentage of the total picture) occupied by the food item (see S1 Fig).

In addition, 24 emotional pictures of negative, positive and neutral valence from the International Affective Picture Set (IAPS, a widely used, standardized database of pictures for studying emotion and attention) were selected according to the population norms provided by the authors (Lang et al., 2005) and ratings by children and adolescents ([30]; IAPS numbers see S1 Table).

The involvement of activity in certain “reward processing” regions depended on attention focus of the participants [16]. Therefore, to keep the subjects’ attention up and to gain information about subjective experiences of the pictures, we asked participants to explicitly focus on the food stimuli and rate their valence. After viewing each picture, participants rated its valence on a 9 point rating scale (“Self Assessment Manikin”, [31]).

### Procedure

Participants were instructed to eat lunch and a small snack, but not to eat or drink anything 1.5 hours before the experiment. Participants were asked to list their food intake since lunch; inpatient AN participants were supervised during lunch and snack intake. Hunger was assessed via a subjective rating scale ranging from 0 (not hungry at all) to 3 (very hungry) prior to the scanning session. Scans were conducted between 4.30 and 6pm.

Images were presented via goggles fitted to the head coil (NordicNeuroLab Visual System®). Pictures of the five picture categories (high-calorie food, low-calorie food, negative, neural, positive) were shown in randomized order for 12 sec each followed by a baseline interval of 12 sec during which a fixation cross was shown. Participants were instructed to look at each picture and afterwards rate its valence via button presses (NordicNeuroLab Response Grips®).

### Image acquisition

All data was acquired using a 12-channel head coil on a 3T high-field scanner (Magnetom Tim Trio, Siemens Healthcare AG, Erlangen, Germany). Anatomical images were acquired using a T_1_-weighted 3D MPRAGE sequence (1 mm isotropic resolution, TR/TE/FA = 1900/2.25/9, Field of view 25.6 cm × 25.6 cm × 25.6 cm). Functional MRI data (depicting Blood Oxygen Level Dependent (BOLD) contrast) were acquired using a multi-slice 2D EPI sequence (1.5 mm × 1.5 mm in plane resolution, 3 mm slice thickness, 0,75 mm interslice gap, TR/TE/FA = 3000/30/90, FOV = 192mm, 128 x 128 matrix). 304 T*2-weighed whole brain volumes were acquired.

### Data analysis and statistics

Behavioral data (subjective valence ratings) were analyzed by using analysis of variance and post hoc two-tailed t tests. Bonferroni correction for the number of tests was applied.

Imaging data analysis, registration, visualization and statistical analyses were performed with the BrainVoyager QX software package (Brain Innovation B.V., Maastricht; The Netherlands). Pre-processing of the data included three-dimensional motion correction, temporal Gaussian smoothing of 4sec, spatial Gaussian smoothing of 4 mm, linear detrending and temporal high pass filtering using 0.01 Hz. To better assign the brain activities to anatomical locations, the individual brains were transformed into standard stereotactic space (Talairach and Tournoux, 1988) and the functional data were co-registered and superimposed with the anatomical data (3-dimensional MPRAGE data set). Talairach coordinates were used to confirm anatomical regions of activation using Talairach Daemon (Research Imaging Center, University of Texas Health Center, San Antonio, TX, USA). Analysis was done with a general linear model with subjects as a random effect. A boxcar-like predictor was used which described the stimulus category (high-calorie food, low-calorie food, positive, neutral, negative). The predictor function was convolved with a haemodynamic response function to consider delays and graded response times of the BOLD signal to the stimuli. For this, a 2γ-haemodynamic response function was used (time to response to peak: 5 s, time to undershoot peak: 15 s). The effects of the predictors on cortical activation were analyzed for AN patients and control participants.

First, a whole-brain group comparison (adolescent patients vs. controls and adult patients vs. controls) was performed for each picture category (see S2 Table for results). The analysis then was restricted to regions of interest (ROIs) which had previously been described as being involved in the processing of food stimuli (see S3 Table).

On the basis of a clustering algorithm, a cortical brain site was regarded as activated only if a minimum cluster size of 150 mm^3^ was reached [32]. The level of significance for the detection of activated cortical areas was determined by Bonferroni correction for the number of clusters analyzed. Only clusters which belonged to one of the ROIs defined were taken for the ongoing analysis. Analyses were performed at the group level (multi-study t tests) for adult and adolescent participants separately. Food stimuli and emotional stimuli were analyzed separately to differentiate disease-specific from general emotion-induced effects. In addition, for food stimuli group comparisons of adult vs. adolescent patients and adult vs. adolescent controls were performed in order to reveal potential developmental effects.

## Results

### Participant characteristics

Participants were between 12.4 and 17.7 (adolescents) and between 18.4 and 40.6 years of age (adults). There were no group differences regarding age for adolescent as well as for adult participants, whereas BMI for adults and BMI age percentile for adolescents were significantly lower for AN patients than for control participants. Both groups rated their feeling of hunger at the beginning of the experimental session similarly. Group differences for ED symptoms assessed by the EDI-2 total score and drive for thinness subscale and depressive symptom scores were larger in adults than in adolescents (see Table 2). Of the adult AN patients, ten were taking antidepressant medication, four antipsychotic medication and one participant antiepileptic medication. Three adolescent AN patients were taking antidepressant medication.

**Table 2:** Participant characteristics and subjective ratings.

Age group	Adolescents (n = 33)			Adults (n = 32)		
Participant group	AN (n = 15)	C (n = 18)	p	AN (n = 16)	C (n = 16)	p
**Demographic characteristics**						
Age (years)	16.41 (1.38)	15.95 (2.12)	n.s. (> .05)	26.71 (6.62)	26.88 (5.60)	n.s. (> .05)
BMI (kg/m^2^)				16.20 (2.02)	21.40 (1.40)	< .001
BMI age percentile	2.87 (3.79)	48.99 (30.69)	< .001			
Hunger feeling	2.07 (0.80)	2.06 (0.57)	n.s. (> .05)	2.13 (0.89)	2.25 (0.58)	n.s. (> .05)
**Clinical characteristics**						
BDI	21.67 (10.97)	10.59 (9.61)	< .01	30.67 (8.36)	4.29 (5.27)	< .001
EDI-2 total score	278.68 (68.26)	225.19 (64.21)	< .05	353.55 (32.66)	209.54 (42.94)	< .001
EDI-2 drive for thinness	28.20 (9.53)	18.65 (9.03)	< .01	31.93 (9.19)	15.94 (4.80)	< .001
AN subtype	13 rAN, 1 bpAN, 1 atypAN			8 rAN, 7 bpAN, 1 atypAN		
Illness duration (months)	14.5 (10.5)			104.8 (98.2)	p < .01 (adolescents vs. adults)	
**Subjective Ratings**						
negative	2.10 (0.66)	2.37 (1.06)	n.s.	2.14 (0.91)	1.79 (0.47)	n.s.
neutral	5.01 (0.93)	4.78 (0.63)	n.s.	5.07 (0.64)	5.24 (0.61)	n.s.
positive	7.47 (0.99)	7.51 (1.09)	n.s.	7.49 (1.01)	7.80 (0.62)	n.s.
low-calorie	6.38 (1.05)	6.82 (0.88)	n.s.	6.97 (1.07)	6.47 (0.75)	n.s.
high-calorie	4.08 (2.20)	6.25 (1.50)	< .01	4.41 (1.78)	5.61 (0.95)	< .05

AN = Anorexia nervosa patients, C = Control participants, rAN = restricting Anorexia nervosa, bpAN = binge-eating/purging type Anorexia nervosa, atypAN = atypical Anorexia nervosa, BMI = Body Mass Index, EDI-2 = Eating Disorder Inventory-2, BDI = Beck Depression Inventory.

### Behavioral data

#### Food stimuli

In an Analysis of variance (ANOVA) with the within-subjects factor “picture category” (high-calorie, low-calorie) and the between-subjects factors “participant group” (AN, controls) and “age group” (adolescents, adults), there was a significant “picture category” effect (*𝐹* (1, 63) = 45.09, p < .001, part. *𝜂^2^* = .43) and “picture category*participant group” interaction (*𝐹* (1, 63) = 13.09, p≤.001, part. *𝜂^2^* = .18). While low-calorie stimuli obtained highest valence ratings by both groups, AN patients rated the high-calorie stimuli less positive than control participants, but low-calorie stimuli similar to controls (see Table 2). Another main effect for “participant group” (*𝐹* (1,63) = 10.87, p < .01, part. *𝜂^2^* = .15) and a significant interaction between “participant group”and “age group”(*𝐹*(1, 63) = 4.04, p < .05, part. *𝜂^2^* = .063) was found with generally lower ratings by patients and a greater group difference in adolescents than in adults. *t* tests, which were conducted for each age group separately, showed a highly significant difference between adolescent patients and controls in valence ratings of high-calorie food stimuli (*𝑡*(30) = 3.27, p≤.01), while this difference between adult patients and controls (*𝑡*(31) = 2.48, p = .020) became non-significant after Bonferroni correction (*𝛼*’ = .0125).

#### Emotional stimuli

For valence ratings, an ANOVA with the within-subjects factor “picture category” (positive, neutral, negative) and the between-subjects factors “participant group” (AN, controls) and “age group” (adolescents, adults) yielded a significant effect of “picture category” (𝐹 (2, 126) = 639.47, p≤.001, part. *η*2 = .91) only. Highest valence ratings from all groups were obtained for positive, followed by neutral and negative stimuli.

### Activation patterns

#### Food stimuli

20 clusters were regarded as activated, which yielded a level of significance of *α*‘ = .0025 after Bonferroni correction.

Group differences in adult participants

Activation differences between AN patients and control participants were revealed predominantly in the cerebellum (increased activation in patients as compared to controls for high- and low-calorie stimuli). When confronted with low-calorie pictures, additional diminished right IFG activation in AN patients was seen (see Table 3).

**Table 3:** Activation differences between adult AN and control participants for food stimuli.

Stimulus category	Brain region	H	Talairach coordinates			BA	Significance
			x	y	z		
High-calorie							
AN>CON							
	Cerebellum	R	28 31	-70 -43	-24 -18	- -	*𝑡* = 5.89, p≤.0001*** 𝑡 = 4.92, p≤.0001***
	Cerebellum	L	-18 -41	-70 -68	-24 -21	- -	*𝑡* = 5.06, p≤.0001*** 𝑡 = 4.50, p≤.0001***
Low-calorie							
AN>CON							
	Cerebellum	R	28 31	-70 -43	-24 -18	-	*𝑡* = 4.82, p≤.0001*** 𝑡 = 3.84, p = .0001**
	Cerebellum	L	-41	-68	-21	-	*𝑡* = 4.69, p≤.0001***
CON>AN							
	Inferior frontal gyrus	R	47	12	28	9	*𝑡𝑡* = -3.19, p = .0001**
	Thalamus	R	25	-31	6	-	*𝑡* = -3.18, p = .0015*

BA = Brodman Area, H = Hemisphere. T score and p value (* = < .05, ** = < .01, *** = < .001 after Bonferroni correction) are provided.

Group differences in adolescent participants

**Fig 1:**
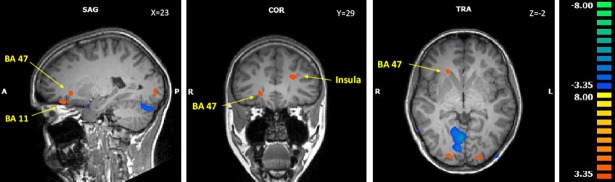
Activation differences between adolescent AN patients and controls for high-calorie stimuli. The color marking shows the t-values of the activation contrast between AN patients and controls. Positive values (red to yellow scale) mark brain areas with higher BOLD changes in AN while negative values (blue to green scale) mark areas with higher BOLD changes in controls.

Viewing high-calorie stimuli elicited stronger IFG, medial prefrontal gyrus and anterior insula activation in adolescent AN participants than in controls (see Fig 1), but diminished activity in the right cerebellum.

For low-calorie stimuli, controls showed increased activity in parts of the right cerebellum, and less left cerebellar, medial prefrontal gyrus and lower parietal lobe activity compared to AN patients (see Table 4).

**Table 4:** Activation differences between adolescent AN and control participants for food stimuli.

Stimulus Category	Brain region	H	Talairach coordinates			BA	Significance
			x	y	z		
High calorie							
AN>CON							
	Inferior frontal gyrus	R	22	28	-2	47	*𝑡* = 3.34, p = .0008**
	Medial prefrontal gyrus	R	24	39	-14	11	*𝑡* = 3.91, p≤.0001**
	Insula	L	-27	26	15	13	*𝑡* = 3.23, p = .0012*
CON>AN							
	Cerebellum	R	5	-64	-2	-	*𝑡* = -4.96, p≤.0001***
Low calorie							
AN>CON							
	Cerebellum	L	-24	-75	-17	-	*𝑡* = 3.25, p = .0012**
	Medial prefrontal gyrus	R	24	39	-14	11	*𝑡* = 4.16, p≤.0001**
	Inferior parietal cortex	R	53	-43	37	40	*𝑡* = 3.47, p = .0005**
CON>AN							
	Cerebellum	R	5 15	-64 -85	-2 -27	- -	*𝑡𝑡* = -4.51, p≤.0001*** 𝑡 = -3.28, p = .0010*

BA = Brodman Area, H = Hemisphere. T score and p value (* = < .05, ** = < .01, *** = < .001 after Bonferroni correction) are provided.

Group comparison adult vs. adolescent patients and adult vs. adolescent controls

In AN patients, direct comparison of adults and adolescents yielded a differential pattern:

Whereas adults showed stronger superior parietal and cerebellum activity when viewing high-calorie stimuli, adolescents showed greater activity in a number of regions (bilateral superior frontal lobe, bilateral cingulate and left cerebellum when viewing low-calorie stimuli, see Table 5).

**Table 5:** Activation differences between adolescent and adult AN patients for food stimuli.

Stimulus Category	Brain region	H	Talairach coordinates			BA	Significance
			x	y	z		
High calorie							
Adults>Adolescents							
	Superior Parietal Lobe	L	-2	-68	56	7	*𝑡* = -3.57, p = .0004**
	Cerebellum	R	36	-74	-16		*𝑡* = -5.77, p < .0001***
Low calorie							
Adolescents>Adults							
	Cingulate	R	2	25	26	32	*𝑡* = 3.64, p = .0003**
	Anterior Cingulate	R	8	35	13	32	*𝑡* = 4.18, p < .0001***
	Anterior Cingulate	L	-3	33	24	32	*𝑡* = 4.01, p < .0001**
	Superior Frontal Lobe	R	5	52	24	9	*𝑡* = 3.70, p = .0002**
	Superior Frontal Lobe	L	-11	51	22	9	*𝑡* = 3.81, p = .0001**
	Cerebellum	L	-27	-71	-15	-	*𝑡* = 4.10, p < .0001***

BA = Brodman Area, H = Hemisphere. T score and p value (** = < .01, *** = < .001 after Bonferroni correction) are provided.

In healthy controls, adolescents’ brain activity in the cingulate, insula and several cerebellar regions was enhanced for high-calorie stimuli compared to adult participants. For low-calorie stimuli this was the case in the caudate, superior frontal gyrus and similar cerebellar regions. Adults showed increased activity compared to adolescents in the left cerebellum for both food stimulus categories (see Table 6).

**Table 6:** Activation differences between adolescent and adult control participants for food stimuli.

Stimulus Category	Brain region	H	Talairach coordinates			BA	Significance
			x	y	z		
High calorie							
Adolescents>Adults							
	Cingulate Gyrus	R	2	25	26	32	*𝑡* = 3.46, p = .0005*
	Insula	R	47	10	-1	13	*𝑡* = 3.75, p≤.0002**
	Cerebellum	R	23	-71	-21	-	*𝑡* = 5.59, p < .0001***
	Cerebellum	L	-24	-71	-26	-	*𝑡* = 5.58, p < .0001***
	Cerebellum	L	-6	-76	-25	-	*𝑡* = 4.42, p < .0001***
Adults>Adolescents							
	Cerebellum	L	0	-32	-26	-	*𝑡* = -3.91, p < .0001***
Low calorie							
Adolescents>Adults							
	Caudate Body	L	-2	3	16	-	*t* = 4.00, p = .0007*
	Inferior Frontal Lobe	L	-24	13	-15	47	*𝑡* = 3.70, p = .0002**
	Cerebellum	R	23	-71	-21	-	*𝑡* = 7.21, p < .0001***
	Cerebellum	L	-24	-71	-26	-	t = 4.87, p≤.0001***
	Cerebellum	L	-6	-76	-25	-	*𝑡* = 4.01, p < .0001***
Adults>Adolescents							
	Cerebellum	L	0	-32	-26	-	*𝑡* = -3.91, p≤.0001***

BA = Brodman Area, H = Hemisphere. T score and p value (* = < .05, ** = < .01, *** = < .001 after Bonferroni correction) are provided.

#### Emotional stimuli

24 clusters were analyzed for group differences, so a level of significance of *α‘* = .00208 was determined after Bonferroni correction.

Group differences in adult participants

Differences in processing of emotional stimuli between adult AN patients and control participants were found in the cerebellum for all three picture categories (positive, neutral, negative). Further group differences involved the inferior frontal gyrus (IFG), with stronger activation when processing negative valenced stimuli in controls (left) and when processing neutral pictures for controls (right). For positive valenced pictures, patients exhibited elevated activation in the right precuneus (see Table 7).

**Table 7:** Activation differences between adult AN and control participants for emotional stimuli.

Stimulus Category	Brain region	H	Talairach coordinates			BA	Significance
			x	y	z		
**negative**							
AN>CON							
	Cerebellum	L	-13	-70	-23	-	*𝑡* = 3.37, p = .0007*
CON>AN							
	Striatum	R	17	-8	28	-	*𝑡* = -3.53, p = .0004*
	Cerebellum	L	-27	-83	-18	-	*𝑡* = -5.34, p≤.0001***
	Cerebellum	R	15	-84	-18	-	*𝑡* = -4.38, p≤.0001***
	Inferior frontal gyrus	L	-59	6	27	9	*𝑡* = -3.51, p = .0004*
**neutral**							
AN>CON							
	Cerebellum	L	-13	-70	-23	-	*𝑡* = 4.15, p≤.0001**
	Cerebellum	R	23	-68	-24	-	*𝑡* = 4.48, p≤.0001***
CON>AN							
	Cerebellum	L	-27	-83	-18	-	*𝑡* = -5.00, p≤.0001***
	Inferior frontal gyrus	R	45	14	29	9	*𝑡* = -3.34, p = .0009*
**positive**							
AN>CON							
	Cerebellum	L	-13	-70	-23	-	*𝑡* = 5.49, p≤.0001***
	Cerebellum	R	23	-68	-24	-	*𝑡* = 4.73, p≤.0001***
	Precuneus	R	21	-62	53	7	*𝑡* = 3.59, p = .0003**
CON>AN							
	Cerebellum	L	-27	-83	-18	-	*𝑡𝑡* = -5.08, p≤.0001***
	Cerebellum	R	15	-84	-18	-	*𝑡* = -3.79, p = .0002**

BA = Brodman Area, H = Hemisphere. T score and p value (* = < .05, ** = < .01, *** = < .001 after Bonferroni correction) are provided.

Group differences in adolescent participants

**Fig 2:**
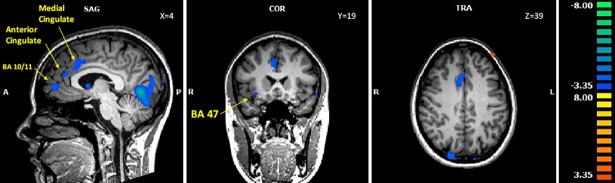
Activation differences between adolescent AN patients and controls for negative stimuli. For details see Fig 1 legend.

In most clusters adolescent control participants showed greater activations than girls suffering from AN. For negative stimuli that was the case in the cerebellum, the ACC, the striatum, frontal and temporal areas (see Fig 2). For neutral pictures this pattern occurred in the cerebellum only and for positive stimuli in regions of the cerebellum and the striatum (caudate and putamen), the ACC, in temporal, parietal and frontal areas as well as in the hippocampus. One region activated stronger in adolescent AN patients than in controls when confronted with negative and neutral stimuli was the right medial prefrontal gyrus (see Table 8).

**Table 8:** Activation differences between adolescent AN and control participants for emotional stimuli.

Stimulus Category	Brain region	H	Talairach coordinates			BA	Significance
			x	y	z		
**negative**							
CON>AN							
	Cerebellum	R	7	-66	-4	-	*𝑡* = -6.64, p≤.0001***
	Anterior Cingulate	R	6	16	40	32	*𝑡* = -3.80, p = .0001**
	Striatum	L	-11	1	12	-	*𝑡* = -4.07, p≤.0001**
	Striatum	R	6	6	8	-	*𝑡* = -4.28, p≤.0001**
	Inferior frontal gyrus	R	41	33	-7	47	*𝑡* = -3.29, p = .0010*
**neutral**							
AN> CON							
	Medial prefrontal gyrus	R	23	41	-15	11	*𝑡* = 3.90, p≤.0001**
CON>AN							
	Cerebellum	R	7	-66	-4	-	*𝑡* = -4.74, p≤.0001***
**positive**							
AN>CON							
	Medial prefrontal gyrus	R	23	41	-15	11	*𝑡* = 3.79, p = .0002**
CON>AN							
	Cerebellum	R	23 7	-68 -66	-24 -4	- -	*𝑡𝑡* = -3.19, p = .0014* 𝑡 = -5.25, p≤.0001***
	Striatum	L	-11	1	12	-	*𝑡* = -4.07, p = .0004*
	Striatum	R	6 25	6 5	8 5	- -	*𝑡* = -4.28, p≤.0001*** 𝑡 = -3.21, p = .0013*
	Anterior cingulate	R	4	23	38	32	*𝑡* = -3.47, p = .0005*
	Precuneus	L	-20	-65	40	7	*𝑡* = -3.57, p = .0003**
	Inferior frontal gyrus	R	41	33	-7	47	*𝑡* = -4.33 p≤.0001***
	Inferior frontal gyrus	L	-41	33	-7	47	*𝑡* = -3.99, p≤.0001**
	Hippocampus	R	27	-17	-16	-	*𝑡* = -4.26 p≤.0001***

BA = Brodman Area, H = Hemisphere. T score and p value (* = < .05, ** = < .01, *** = < .001 after Bonferroni correction) are provided.

Group comparison adult vs. adolescent patients and adult vs. adolescent controls

Comparison of adult and adolescent AN patients’ activation patterns for emotional stimuli showed hyperactivations of the cerebellum in adults vs. adolescents for negative stimuli, but in adolescents vs. adults for neutral and positive stimuli. Superior parietal areas and different frontal areas (superior, middle, inferior) were more active in adults for positive stimuli (see Table 9). In control participants, apart from cerebellar activity, where again a mixed pattern was seen, mainly hyperactivations in adolescents vs. adults were detected: The cingulate (for neutral and negative stimuli), the caudate (for positive and negative stimuli), the insula and inferior and middle frontal regions (only for negative stimuli) showed increased activity in the younger control subgroup (see Table 10).

**Table 9:** Activation differences between adolescent and adult AN patients for emotional stimuli.

Stimulus Category	Brain region	H	Talairach coordinates			BA	Significance
			x	y	z		
**Negative**							
Adolescents>Adults							
	Cerebellum	L	-27	-71	-15	-	*𝑡* = 3.65, p = .0003**
	Cerebellum	R	31	-53	-19	-	*𝑡* = 3.87, p = .0001**
**Neutral**							
Adults>Adolescents							
	Cerebellum	R	36	-74	-16	-	*𝑡* = -5.00, p < .0001***
	Cerebellum	L	-38	-79	-16	-	*𝑡* = -4.00, p < .0001**
	Cerebellum	L	-6	-76	-25	-	*𝑡* = -3.77, p = .0002**
**Positive**							
Adults>Adolescents							
	Superior Parietal Lobe	L	-2	-68	56	7	*𝑡* = -3.65, p = .0003**
	Superior Parietal Lobe	R	19	-67	54	7	*𝑡* = -5.12, p < .0001***
	Superior Frontal Lobe	R	20	63	0	10	*𝑡* = -3.81, p = .0001**
	Superior Frontal Lobe	L	-19	64	12	10	*𝑡* = -4.49, p < .0001***
	Inferior Frontal Lobe	L	-41	28	-4	47	*𝑡* = -4.17, p < .0001**
	Inferior Frontal Lobe	R	32	14	-19	47	*𝑡* = -3.75, p = .0002**
	Inferior Frontal Lobe	L	-42	51	-1	10	*𝑡* = -4.19, p < .0001**
	Middle Frontal Lobe	R	48	52	4	10	*𝑡* = -4.51, p < .0001***
	Caudate Body	R	7	17	14	-	*𝑡* = -3.85, p = .0001**
	Cerebellum	R	36	-74	-16	-	*𝑡* = -5.96, p < .0001***
	Cerebellum	L	-38	-79	-16	-	*𝑡* = -4.12, p < .0001**
	Cerebellum	L	-6	-76	-25	-	*𝑡* = -4.10, p < .0001**

BA = Brodman Area, H = Hemisphere. T score and p value (** = < .01, *** = < .001 after Bonferroni correction) are provided.

**Table 10:** Activation differences between adolescent and adult control participants for emotional stimuli.

Stimulus Category	Brain region	H	Talairach coordinates			BA	Significance
			x	y	z		
**Negative**							
Adolescents>Adults							
	Cingulate	R	2	25	26	32	*𝑡* = 3.48, p = .0005*
	Cingulate	R	1	23	37	32	*𝑡* = 4.10, p < .0001**
	Cingulate	L	0	-10	34	24	*𝑡* = 3.61, p = .0003**
	Insula	R	47	10	-1	13	*𝑡* = 3.44, p = .0006*
	Caudate Body	L	-11	2	12	-	*𝑡* = 4.62, p < .0001***
	Caudate Body	R	9	3	23	-	*𝑡* = 4.05, p < .0001**
	Medial Frontal Lobe	R	2	45	33	9	*𝑡* = 3.71, p = .0002**
	Medial Frontal Lobe	R	3	52	6	10	*𝑡* = 3.54, p = .0004*
	Frontal Lobe	R	53	12	3	44	*𝑡* = 3.08, p = .0001**
	Inferior Frontal Lobe	L	-48	18	1	47	*𝑡* = 3.60, p = .0003**
	Cerebellum	L	-9	-71	-22	-	*𝑡* = 6.37, p < .0001***
	Cerebellum	L	-24	-71	-26	-	*𝑡* = 5.22, p < .0001***
	Cerebellum	R	23	-71	-22	-	*𝑡* = 4.96, p < .0001***
	Cerebellum	R	31	-53	-19	-	*𝑡* = 4.11, p < .0001**
Adults>Adolescents							
	Cerebellum	L	0	-32	-26	-	*𝑡* = -3.93, p < .0001**
**Neutral**							
Adolescents>Adults							
	Cingulate	R	2	25	26	32	*𝑡* = 3.80, p = .0001**
	Cerebellum	L	-27	-68	-23	-	*𝑡* = 3.71, p = .0002**
	Cerebellum	L	-9	-71	-22	-	*𝑡* = 6.37, p < .0001***
	Cerebellum	R	27	-75	-25	-	*𝑡* = 4.07, p < .0001**
**Positive**							
Adolescents>Adults							
	Caudate Body	R	9	3	23	-	*𝑡* = 3.74, p = .0002**
	Caudate Body	L	-7	8	8	-	*𝑡* = 4.12, p < .0001**
	Cerebellum	L	-9	-71	-22	-	*𝑡* = 6.37, p < .0001***
	Cerebellum	R	23	-71	-22	-	*𝑡* = 4.14, p < .0001**
	Cerebellum	L	-24	-71	-26	-	*𝑡* = 4.13, p < .0001**
Adults>Adolescents							
	Cerebellum	L	0	-32	-26	-	*𝑡* = -4.86, p < .0001***

BA = Brodman Area, H = Hemisphere. T score and p value (* = < .05, ** = < .01, *** = < .001 after Bonferroni correction) are provided.

## Discussion

### Processing of food stimuli

The analysis of activation patterns when viewing food pictures highlighted a few regions with a differential pattern in adults and adolescents. In adult patients, parts of the cerebellum seem to be stronger activated for all food stimuli. In the adolescent sample, AN patients show partly stronger, but mostly weaker cerebellum activity. The cerebellum has recently been examined in the context of eating and feeding behavior [8, 33, 34]. Different cerebellar regions seem to contribute to food related processing: a similar mid-cerebellar region as activated in our study in controls to a greater extent than in AN patients was activated in healthy participants specifically for high-calorie food items, possibly reflecting the integration of sensory and visceral signals [22]. However, mostly widespread areas of the cerebellum show altered activations in AN patients [14], interpreted e.g. as increased appetite response. But the exact mechanisms of the different subsections of the cerebellum including cerebello-cerebral circuits (e.g. to the hypothalamus or limbic system) are not fully understood, so it is unclear why different regions show a different pattern in our results [33]. While currently ill and recovered AN participants showed increased activation of two cerebellar regions after an overnight fast [14], decreased food viewing activation in patients who just ate was found [17], so differences in nutritional status might play a role. However, in our study all participants were asked to take in full meals (intake was supervised in AN patients) and hunger ratings didn’t show any difference between AN patients and controls. Activation patterns being similar regarding emotional and food stimuli, an alternative interpretation could be the association with non-specific processes, such as attention and arousal [9]. This is supported by a similarly unequivocal pattern of cerebellar activation when comparing adolescents and adults suffering from AN on the one hand and healthy girls and adults on the other hand. The cerebellum again showed seemingly non-specific activation differences in both directions.

In the framework of TD and BU areas involved in executive function and control vs. heightened salience attribution and emotional involvement, regions like the OFC or medial frontal cortex are defined as TD areas in some studies [14], in others more as related to the BU reward system and nonhomeostatic food motivation [5, 9]. In our study, activation differences in inferior and medial frontal areas were limited to exposure with low-calorie food in adults, whereas adolescent AN patients showed hyperactivation for food pictures as compared to healthy adolescents in regions belonging to the OFC. Similar areas were more strongly activated when viewing food as opposed to neutral pictures in AN patients compared to controls [8, 9, 19]. The OFC is involved in hedonic food processing, but can be seen in the broader context of decision making, representing the affective value of reinforcers and signaling the expected–positive or negative–outcomes of a situation [35]. In a study with healthy participants, adolescents showed significantly less medial prefrontal response to high-calorie food images than adults with significant age-related increases in the activation of the OFC within the adolescent group [21]. Children and younger adolescents prefer the flavors of foods that are calorie-rich [36], often without engaging in extensive evaluation of their potential rewarding or “punishing” effects like weight-gain. The adolescent AN patients in our study are likely to suffer to a greater extent than their same-age peers from an approach/avoidance conflict when confronted with such food stimuli, which could explain the larger OFC activation differences in adolescents than in adults.

The insula, which serves as a “hub” between TD and BU areas [17] and has been shown to be involved in a network spanning across BU, TD and visual processing areas [14], seems to play a role specifically in adolescent AN patients. Results of increased insula activation in AN “suggest an overly sensitive brain taste reward system” [13, 18, 37]. The left insula also seems to be involved in interoception (it has been linked to inadequately “feeling full”) and hunger regulation [38]. Killgore et al. (2005) found higher insula activation in adolescents than in adults in response to viewing high-calorie foods. This in line with our results where healthy adolescents showed increased insula activation in the high-calorie condition compared to adult controls. In younger adults, as opposed to older adults, the agranular insula, a part of the anterior insula, seems to be related to subjective unpleasantness of taste [39]. Therefore, this part of the taste reward system which underlies changes during adolescent and early adult development seems to be particularly active in adolescent girls suffering from AN. In adolescent control participants, we also found increased cingulate activity compared to adults for high-calorie food viewing, similar to increased activity in this region which was found in another study in hungry healthy adolescents [40].

In patients, however, a similar developmental effect (increased cingulate activation in adolescents vs. adults) was confined to low-calorie stimuli. Therefore motivational processes, which could be increased in the adolescent population in general, seem to extend to different kinds of food: whereas appetitive reactions towards high-calorie food seem natural in healthy adolescents they are unlikely to be present in those suffering from AN. Taken together, our results point more towards hyperactivity of BU as well as TD areas in adolescents suffering from AN, corresponding with hypothesis H1. No such differences were found in adult patients for high-calorie stimuli.

In line with previous studies, AN patients rated high-calorie food stimuli as less pleasant than did controls [4, 5, 9, 41]. In our sample, this group difference was greater in adolescents similar to greater aberration of cerebral activation patterns involving food relevant areas although firm conclusions about ratings correlating with fMRI responses cannot be drawn.

### Processing of emotion eliciting stimuli

Contrary to our hypothesis H2 and unlike two studies which found no specific brain response patterns to emotionally aversive stimuli in AN [10, 24], our results yielded many brain regions which were differently involved in AN patients and controls. Specifically, cerebellar regions were less activated in adolescent AN patients than in controls; within the adult group a mixed pattern emerged in AN patients with increased activity in some subregions and decreased activity in others. Cerebellar hypo- and hyperactivations without a clear pattern throughout all stimulus categories including neutral stimuli–similar to [9]–probably reflect rather non-specific correlates of general emotional attention or arousal. Reduced involvement of emotion processing regions in adolescent AN patients as compared to their typically developing counterparts extends to the ACC, the striatum, frontal and temporal regions (for negative stimuli), and in addition to the precuneus and hippocampus for positive stimuli. Lower ACC activity has already been observed in the context of high alexithymia scores in AN patients when confronted with negative words [23] and might therefore reflect impaired emotional awareness. Similarly, the striatum has been linked to motivational aspects of emotion processing [42]. Our results suggest that precuneus hypoactivation is restricted to positive stimulus processing in AN adolescents. Hagele et al. found activation of the precuneus in an emotional pictures task for pleasant stimuli only [43]. Underactivation in this region therefore contributes to the idea of reduced processing of positive non-specific stimuli. A relatively high hippocampal activity in adolescents as compared to adults was seen in a recent study on processing of emotional pictures throughout adolescence [44]. However, in our study when comparing adult vs. adolescent patients and control participants, there were no group differences regarding hippocampal activity. When processing negative, but partly also positive pictures, healthy adolescents showed higher activity in the cingulate, insula and caudate than their adult counterparts, as postulated in models of relatively early maturation of subcortical areas in adolescence [45]. However, a proposed relatively late maturation of (pre-)frontal areas in typical development [45] is not in line with our results where enhanced frontal activity was also shown in adolescents for negative stimuli, possibly due to enhanced recruitment when coping with disturbing pictures. In AN patients, however, frontal hypoactivations were present in adolescents compared to adults for positive stimuli. In a previous study with healthy adolescents the ventrolateral prefrontal cortex (Brodman Area (BA) 47) was showing increasing activity with age for emotional picture processing [44]. In our study activity in this region appeared to be diminished for adolescent and adult AN patients compared to their healthy counterparts throughout different picture categories. Thus, young AN patients seem to be specifically vulnerable to functional abnormalities concerning regions which are still developing (see hypothesis H3).

Taken together, in adults group differences were observed in fewer regions and in the direction of hypo- as well as hyperactivation in AN patients, so it remains unclear whether and in which respect adult patients are predisposed to emotional vulnerability in general. In AN adolescents, results point towards reduced processing of all emotional stimuli with some specific regions involved in positive picture processing, possibly reflecting reduced ability to experience pleasure by daily natural reinforcers. Importantly, hypoactivity in such brain regions as the OFC [46] and abnormalities in striatum activation [47] have also been found in depression. Depression scores in our study were higher in AN patients, however, the difference was even greater in adults than in adolescents as opposed to the difference regarding neural activations. Furthermore, no differences regarding subjective ratings of picture valence were found in our study.

Adolescent brains are still in a phase of maturation, which refers mainly to frontal regions as opposed to subcortical regions which develop earlier in childhood [45]. An imbalance between those two clusters could affect stimulus processing. Gray and white matter volume loss during acute AN are even more pronounced in adolescence than in adulthood [48]. Although brain volume change in AN seems to be a global phenomenon, especially cerebellar gray matter volume reduction was more often reported for adolescents than for adults. So our results with greater group differences in adolescents for emotional stimuli could partly be related to this phenomenon. However, correlational analyses with structural data would be necessary.

### Limitations

A limitation of the study is the relatively small sample size–although comparable to other fMRI studies–which doesn’t permit us to look at distinct AN subgroups which were different in adult and adolescent patients. A longer illness duration in adults makes it difficult to disentangle effects of age and disease chronicity. Food intake prior to the scan, but not handedness and hydration status were assessed. IAPS pictures are a widely recognised tool with respect to emotion research; however, they differ from the food pictures in this study regarding colour, background etc., so results are not entirely comparable. Also, emotional pictures, e.g. showing social situations, could trigger different emotions in AN patients than in healthy participants. The food items displayed were mostly items considered by healthy persons as pleasurable food which might have affected ratings and activation patterns. The relatively long picture presentation of 12 sec might have caused decreased attention over time and therefore altered BOLD response. Conclusions about developmental trajectories must be made with caution as an adolescent group–irrespective of pubertal stage–has been compared to an adult group and no longitudinal measurements were conducted.

### Conclusions

In our study examining neural responses of AN patients of two age groups, we found a differential pattern for food compared to generally emotion eliciting stimuli. While less clear and specific activation differences were present in adults, adolescents show reduced processing of affective stimuli and enhanced activation of BU and TD areas as well as the insula with regard to food stimuli. Regions which undergo maturational changes during adolescent development seem to be especially vulnerable for altered activation during stimulus processing in AN.

Increasing knowledge about the neurobehavioural mechanisms underlying the perception of food and emotion will lead to practical strategies to deal with anorexia nervosa and related disorders. Behavioral therapy programs could benefit from additional elements addressing e.g. the issues of food craving vs. intake control and emotion regulation. Further research could e.g. focus on the influence of hormonal or neuropeptide aberrations on stimulus processing [49] or on emotion and attention regulation strategies which could help patients coping with confrontation with emotionally salient stimuli.

## Supporting information

S1 Fig High-calorie and low-calorie food stimuli. (TIF)

S1 Table IAPS stimuli. IAPS numbers of positive, neutral and negative pictures.
(DOCX)

S2 Table Results from the whole-brain group comparison (patients vs. controls). Regions with group differences are listed.
(DOCX)

S3 Table Regions of interest. Selected regions of interest based on previous studies on processing of emotional and food stimuli in EDs and in the developing brain.
(DOCX)
