# Supplementary figures and images for: Neural processing of food and emotional stimuli in adolescent and adult anorexia nervosa patients
Source: PLoS One. 2018 Mar 26;13(3):e0191059. doi: 10.1371/journal.pone.0191059 (PMC5868769; doi:10.1371/journal.pone.0191059)

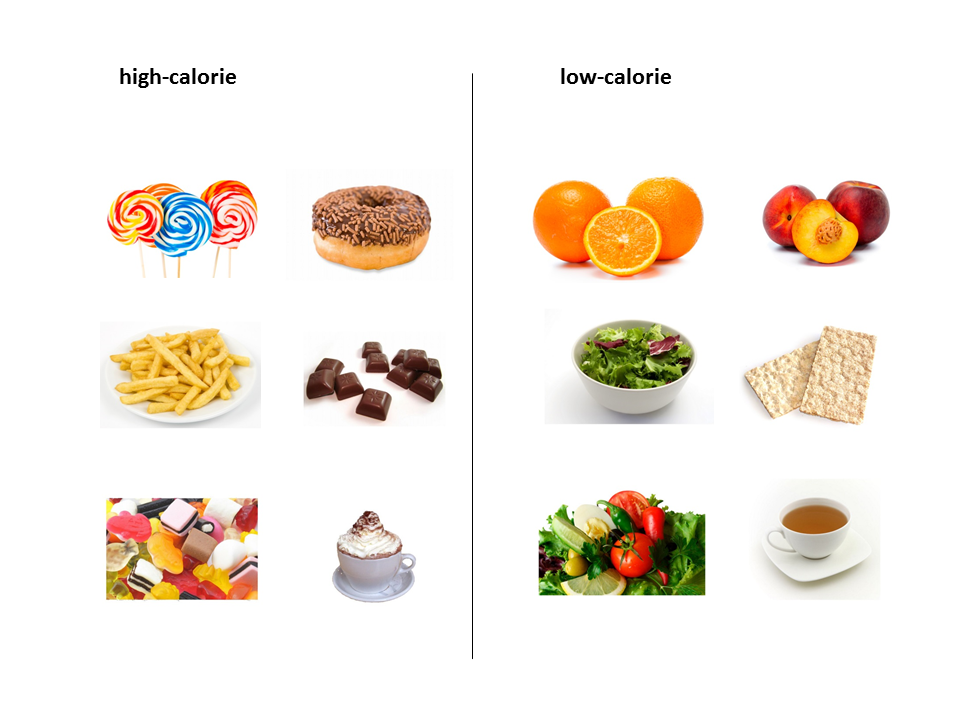

Supplement: S1 Fig — (TIF) [file pone.0191059.s001.tif]
