# Supplementary material for: Neural processing of food and emotional stimuli in adolescent and adult anorexia nervosa patients
Source: PLoS One. 2018 Mar 26;13(3):e0191059. doi: 10.1371/journal.pone.0191059 (PMC5868769; doi:10.1371/journal.pone.0191059)
Supplement: S1 Table — IAPS numbers of positive, neutral and negative pictures. (DOCX) [file pone.0191059.s002.docx]

**S1 Table. IAPS stimuli.** IAPS numbers of positive, neutral and negative pictures.

| **Stimulus category** | **IAPS numbers** |
| --- | --- |
| positive | 1710, 2071, 4542, 5833, 5910, 8370, 8531, 8540 |
| neutral | 2102, 2512, 5740, 5531, 7002, 7009, 7052, 7235 |
| negative | 1120, 2095, 2278, 2682, 2811, 6510, 9340, 9600 |
