# Supplementary material for: Neural processing of food and emotional stimuli in adolescent and adult anorexia nervosa patients
Source: PLoS One. 2018 Mar 26;13(3):e0191059. doi: 10.1371/journal.pone.0191059 (PMC5868769; doi:10.1371/journal.pone.0191059)
Supplement: S2 Table — Regions with group differences are listed. (DOCX) [file pone.0191059.s003.docx]

**S2 Table. Results from the whole-brain group comparison (patients vs. controls).** Regions with group differences are listed.

| **Stimulus category** | **Brain region** | **Talairach coordinates** | **H** | **BA** |
| --- | --- | --- | --- | --- |
| **High-calorie** |  |  |  |  |
| Adults |  |  |  |  |
|  | Middle Frontal Gyrus | -27 8 49 | L | 6 |
|  | Middle Frontal Gyrus | 32 13 48 | R | 6 |
|  | Precentral Gyrus | -43 -2 37 | L | 6 |
|  | Precuneus | -20 -83 37 | L | 19 |
|  | Occipital Lobe | -4 -83 9 | L | 17 |
|  | Occipital Lobe | 19 -86 -2 | R | 17 |
|  | Occipital Lobe | -24 -86 -2 | L | 18 |
|  | Superior Temporal Gyrus | 48 3 -9 | R | 38 |
|  | Frontal Lobe | 6 2 -18 | R | 25 |
|  | Cerebellum | 28 -70 -24 | R |  |
|  | Cerebellum | -18 -70 -24 | L |  |
|  | Medial Frontal Gyrus | -5 -1 54 | L | 6 |
|  | Cerebellum | 31 -43 -18 | R |  |
|  | Cerebellum | -36 -48 -20 | L |  |
|  | Precentral Gyrus | -43 -2 37 | L | 6 |
|  | Precuneus | -20 -83 37 | L | 19 |
|  | Occipital Lobe | -4 -83 9 | L | 17 |
|  | Occipital Lobe | 19 -86 -2 | R | 17 |
|  | Occipital Lobe | -24 -86 -2 | L | 18 |
|  | Superior Temporal Gyrus | 48 3 -9 | R | 38 |
|  | Frontal Lobe | 6 2 -18 | R | 25 |
|  | Cerebellum | 28 -70 -24 | R |  |
|  | Cerebellum | -18 -70 -24 | L |  |
|  | Medial Frontal Gyrus | -5 -1 54 | L | 6 |
|  | Cerebellum | 31 -43 -18 | R |  |
|  | Cerebellum | -41 -68 -21 | L |  |
| Adolescents |  |  |  |  |
|  | Precentral Gyrus | -33 -13 64 | L | 6 |
|  | Frontal Lobe | 27 3 57 | R | 6 |
|  | Precentral Gyrus | -42 1 31 | L | 6 |
|  | Middle frontal Gyrus | -44 21 30 | L | 9 |
|  | Middle frontal Gyrus | -24 32 24 | L | 9 |
|  | Cingulate Gyrus | -2 7 23 | L | 24 |
|  | Anterior Cingulate | 3 9 23 | R | 33 |
|  | Occipital Lobe | 30 -84 20 | R | 19 |
|  | Occipital Lobe | -15 -84 26 | L | 18 |
|  | Cerebellum | 5 -64 -2 | R |  |
|  | Inferior Frontal Gyrus | 22 28 -2 | R | 47 |
|  | Superior Temporal Gyrus | 23 62 -2 | R | 10 |
|  | Parahippocampal Gyrus | -42 -46 -2 | L | 19 |
|  | Inferior Frontal Gyrus | -19 28 -3 | L | 47 |
|  | Medial Frontal Gyrus | 24 39 -14 | R | 11 |
|  | Parahippocampal Gyrus | 19 4 -20 | R | 34 |
|  | Insula | -27 26 15 | L | 13 |
| **Low calorie** |  |  |  |  |
| Adults |  |  |  |  |
|  | Inferior Frontal Gyrus | 47 12 29 | R | 9 |
|  | Inferior Frontal Gyrus | -58 6 26 | L | 9 |
|  | Insula | -45 -38 22 | L | 13 |
|  | Anterior Cingulate | 9 32 13 | R | 24 |
|  | Occipital Lobe | -5 -84 12 | L | 17 |
|  | Occipital Lobe | -42 -85 7 | L | 19 |
|  | Superior Frontal Gyrus | -22 52 3 | L | 10 |
|  | Occipital Lobe | 20 -85 1 | R | 17 |
|  | Lateral Pallidum | 16 2 -9 | R |  |
|  | Cerebellum | 31 -43 -18 | R |  |
|  | Cerebellum | 28 -70 -24 | R |  |
|  | Cerebellum | -41 -68 -21 | L |  |
|  | Thalamus | 25 -32 6 | R |  |
| Adolescents |  |  |  |  |
|  | Postcentral Gyrus | -10 -37 61 | L | 4 |
|  | Middle Frontal Gyrus | -32 38 37 | L | 9 |
|  | Inferior Parietal Lobe | 53 -43 37 | R | 40 |
|  | Inferior Parietal Lobe | -55 -44 38 | L | 40 |
|  | Cingulate Gyrus | -3 -37 31 | L | 31 |
|  | Superior Frontal Gyrus, | -12 56 22 | L | 9 |
|  | Anterior Cingulate | -11 35 21 | L | 32 |
|  | Inferior Frontal Gyrus | -49 16 19 | L | 44 |
|  | Occipital Lobe | 10 -80 19 | R | 18 |
|  | Occipital Lobe | -17 -82 25 | L | 18 |
|  | Occipital Lobe | 31 -83 17 | R | 19 |
|  | Posterior Cingulate | 14 -48 12 | R | 29 |
|  | Posterior Cingulate | -13 -49 13 | L | 30 |
|  | Anterior Cingulate | 12 29 17 | R | 24 |
|  | Anterior Cingulate | -14 36 19 | L | 32 |
|  | Inferior frontal gyrus | -49 15 16 | L | 44 |
|  | Insula | 39 19 16 | R | 13 |
|  | Thalamus | -6 -7 9 | L |  |
|  | Thalamus | 17 -34 10 | R |  |
|  | Posterior Cingulate | 14 -47 11 | R | 29 |
|  | Posterior Cingulate | -14 -50 12 | L | 30 |
|  | Cerebellum | 5 -64 -2 | R |  |
|  | Occipital Lobe | 7 -79 0 | R | 18 |
|  | Occipital Lobe | 19 -90 -1 | R | 17 |
|  | Occipital Lobe | -19 -91 0 | L | 17 |
|  | Medial Frontal Gyrus | 24 39 -14 | R | 11 |
|  | Cerebellum | -24 -75 -17 | L |  |
|  | Cerebellum | 15 -85 -27 | R |  |
| **Negative** |  |  |  |  |
| Adults |  |  |  |  |
|  | Parietal Lobe | 43 -29 45 | R | 40 |
|  | Cingulate Gyrus | 19 -38 37 | R | 31 |
|  | Caudate Body | 17 -8 28 | R |  |
|  | Caudate Body | -18 -18 28 | L |  |
|  | Inferior Frontal Gyrus | -59 6 27 | L | 9 |
|  | Precentral Gyrus | 60 2 26 | R | 6 |
|  | Middle Frontal Gyrus | 46 44 25 | R | 46 |
|  | Middle Frontal Gyrus | -47 40 25 | L | 46 |
|  | Insula | -42 -39 24 | L | 13 |
|  | Caudate Tail | 17 -31 23 | R |  |
|  | Thalamus | -11 -31 20 | L |  |
|  | Superior Frontal Gyrus | 39 37 17 | R | 10 |
|  | Claustrum | 28 13 17 | R |  |
|  | Inferior Frontal Gyrus | 45 19 11 | R | 45 |
|  | Parahippocampal Gyrus | -27 -49 10 | L | 30 |
|  | Occipital Lobe | -9 -77 2 | L | 18 |
|  | Occipital Lobe | 47 -69 1 | R | 37 |
|  | OccipitalLobe | -40 -84 6 | L | 19 |
|  | Posterior Cingulate | 20 -65 5 | R | 30 |
|  | Putamen | -29 6 -2 | L |  |
|  | Occipital Lobe | 9 -73 -4 | R | 18 |
|  | Cerebellum | -27 -83 -18 | L |  |
|  | Cerebellum | -13 -70 -23 | L |  |
|  | Cerebellum | 15 -84 -18 | R |  |
| Adolescents |  |  |  |  |
|  | Anterior Cingulate | 6 16 40 | R | 32 |
|  | Caudate Body | -11 1 12 | L |  |
|  | Occipital Lobe | -14 -83 24 | L | 18 |
|  | Occipital Lobe | 11 -80 22 | R | 18 |
|  | Caudate Body | -9 3 10 | L |  |
|  | Caudate Body | 6 6 8 | R |  |
|  | Occipital Lobe | -18 -92 1 | L | 17 |
|  | Occipital Lobe | 17 -90 -2 | R | 17 |
|  | Occipital Lobe | 7 -67 0 | R | 19 |
|  | Inferior Frontal Gyrus | 41 33 -7 |  |  |
|  | Middle Frontal Gyrus | 46 3 51 | R | 6 |
|  | Medial Frontal Gyrus | 4 51 7 | R | 10 |
|  | Superior Frontal Gyrus | 27 62 1 | R | 10 |
|  | Superior Frontal Gyrus | -31 59 -2 | L | 10 |
|  | Superior Temporal Gyrus | 54 13 -3 | R | 22 |
|  | Cerebellum | 7 -66 -4 | R |  |
| **Neutral** |  |  |  |  |
| Adults |  |  |  |  |
|  | Inferior Frontal Gyrus | 45 14 29 | R | 9 |
|  | Superior Frontal Gyrus | -10 69 72 | L | 10 |
|  | Occipital Lobe | 16 -69 21 | R | 18 |
|  | Middle Frontal Gyrus | 39 49 13 | R | 10 |
|  | Occipital Lobe | -6 -83 10 | L | 17 |
|  | Occipital Lobe | -42 -84 6 | L | 19 |
|  | Occipital Lobe | 18 -84 -2 | R | 18 |
|  | Hippocampus | -31 -20 -7 | L |  |
|  | Fusiform Gyrus | 33 -41 -16 | R | 20 |
|  | Cerebellum | 23 -68 -24 | R |  |
|  | Cerebellum | -27 -83 -18 | L |  |
|  | Cerebellum | -13 -70 -23 | L |  |
| Adolescents |  |  |  |  |
|  | Middle Frontal Gyrus | 44 14 35 | R | 9 |
|  | Cingulate Gyrus | 6 32 27 | R | 32 |
|  | Middle Frontal Gyrus | 37 40 14 | R | 10 |
|  | Middle Frontal Gyrus | -39 48 19 | L | 46 |
|  | Occipital Lobe | 26 -80 15 | R | 18 |
|  | Occipital Lobe | -18 -81 22 | L | 18 |
|  | Occipital Lobe | 8 -83 10 | R | 17 |
|  | Fusiform Gyrus | 40 -62 -7 | R | 37 |
|  | Middle Frontal Gyrus | -30 50 -11 | L | 11 |
|  | Middle Frontal Gyrus | 31 49 -12 | R | 11 |
|  | Hypothalamus | -3 -6 -17 | L |  |
|  | Superior Frontal Gyrus | 23 41 -15 | R | 8 |
|  | Cerebellum | 7 -66 -4 | R |  |
| **Positive** |  |  |  |  |
| Adult |  |  |  |  |
|  | Superior Frontal Gyrus | 25 39 51 | R | 8 |
|  | Medial Frontal Gyrus | -5 -3 55 | L | 6 |
|  | Precuneus | 21 -62 53 | R | 7 |
|  | Middle Frontal Gyrus | -47 10 45 | L | 6 |
|  | Precentral Gyrus | -59 -13 35 | L | 4 |
|  | Superior Frontal Gyrus | -30 52 23 | L | 10 |
|  | Occipital Lobe | -5 -80 9 | L | 17 |
|  | Occipital Lobe | 30 -73 16 | R | 30 |
|  | Occipital Lobe | 18 -82 -4 | R | 18 |
|  | Occipital Lobe | 43 -71 -4 | R | 19 |
|  | Cerebellum | 15 -84 -18 | R |  |
|  | Cerebellum | -27 -83 -18 | L |  |
|  | Cerebellum | -13 -70 -23 | L |  |
|  | Cerebellum | 23 -68 -24 | R |  |
|  | Superior Temporal Gyrus | -56 1 0 | L | 22 |
|  | Subcallosal Gyrus | 8 23 -16 | R | 25 |
|  | Hypothalamus | 8 -6 -20 | R |  |
|  | Superior Temporal Gyrus | 41 6 -24 | R | 38 |
|  | Fusiform Gyrus | 36 -41 -18 | R | 20 |
| Adolescents |  |  |  |  |
|  | Medial Frontal Gyrus | 0 47 40 | L | 8 |
|  | Anterior Cingulate | 4 23 38 | L | 32 |
|  | Precuneus | -20 -65 40 | L | 7 |
|  | Precuneus | 23 -60 36 | R | 7 |
|  | Superior Frontal Gyrus | -11 67 16 | L | 10 |
|  | Anterior Cingulate | -1 40 11 | L | 32 |
|  | Caudate Body | 6 6 8 | R |  |
|  | Caudate Body | -11 1 12 | L |  |
|  | Putamen | 25 5 5 | R |  |
|  | Occipital Lobe | 4 -84 15 | R | 18 |
|  | Thalamus | 0 -12 18 | L |  |
|  | Putamen | -24 -6 7 | L |  |
|  | Superior Frontal Gyrus | 27 54 4 | R | 10 |
|  | Medial Frontal Gyrus | 3 58 5 | R | 10 |
|  | Superior Frontal Gyrus | -24 51 -1 | L | 10 |
|  | Middle Frontal Gyrus | 33 51 -1 | R | 10 |
|  | Inferior Frontal Gyrus | 41 33 -7 | R | 47 |
|  | Inferior Frontal Gyrus | -41 33 -7 | L | 47 |
|  | Cerebellum | 23 -68 -24 | R |  |
|  | Cerebellum | 7 -66 -4 | R |  |
|  | Occipital Lobe | -23 -73 -5 | L | 18 |
|  | Middle Temporal Gyrus | 61 -26 -8 | R | 21 |
|  | Middle Temporal Gyrus | -62 -30 -9 | L | 21 |
|  | Superior Frontal Gyrus | 23 41 -15 | R | 11 |
|  | Hippocampus | 27 -17 -16 | R |  |
|  | Temporal Lobe | -36 -17 -22 | L | 20 |
|  | Superior Temporal Gyrus | 54 13 -3 | R | 22 |
|  | Fusiform Gyrus | -57 -10 -24 | L | 20 |

BA=Brodman Area, H=Hemisphere.
