# Supplementary material for: Neural processing of food and emotional stimuli in adolescent and adult anorexia nervosa patients
Source: PLoS One. 2018 Mar 26;13(3):e0191059. doi: 10.1371/journal.pone.0191059 (PMC5868769; doi:10.1371/journal.pone.0191059)
Supplement: S3 Table — Selected regions of interest based on previous studies on processing of emotional and food stimuli in EDs and in the developing brain. (DOCX) [file pone.0191059.s004.docx]

**S3 Table. Regions of Interest.** Selected Regions of Interest based on previous studies on processing of emotional and food stimuli in EDs and in the developing brain.

| **Emotional stimuli (hypotheses H2 and H3)** | **Food stimuli (hypotheses H1 and H3)** |
| --- | --- |
| 1. Precuneus: BA 7 (Joos et al., 2011;  Masumoto et al., 2006) | 1.Inferior frontal gyrus/ Orbitofrontal cortex: BA 9, BA 44,BA 47 (Holsen et al., 2012; Killgore and Yurgelun-Todd, 2005; Kim et al.,  2012; Uher et al., 2003) |
| 2. Inferior frontal gyrus: BA 9, BA 47  (Miyake et al., 2012) |  |
|  | 2. Medial prefrontal gyrus/Orbitofrontal cortex: BA 11 (Frank et al., 2012; Uher et al., 2004; Miyake et al., 2012) |
| 3. Medial prefrontal gyrus: BA 11  (Uher et al., 2004; Miyake et al., 2012) |  |
|  | 3. Insula: BA 13 (Brooks et al., 2012;  Holsen et al., 2012) |
| 4. Insula: BA 13 (Miyake et al., 2012) |  |
| 5. Anterior cingulate cortex: BA 32 (Kim et al., 2012; Uher et al., 2003) | 4. Anterior cingulate cortex: BA 24, BA 32, BA 33 (Kim et al., 2012; Uher et al., 2003) |
| 6. Inferior parietal cortex: BA 40 (Miyake  et al., 2012; Uher et al., 2004) | 5. Inferior parietal cortex: BA 40 (Santel,  Baving, et al., 2006; Uher et al., 2004) |
| 7. Cerebellum (Uher et al., 2004) | 6. Cerebellum (Brooks et al., 2012; Kim et  al., 2012; Uher et al., 2004) |
| 8. Hippocampus (Holsen et al., 2012; Vink et al., 2014) | 7. Hippocampus (Holsen et al., 2012) |
|  | 8. Amygdala (Holsen et al., 2012; Joos et al., 2011) |
|  | 9. Striatum (Sanders et al., 2015) |
